# Automated Segmentation of Levator Ani Muscle from 3D Endovaginal Ultrasound Images

**DOI:** 10.3390/bioengineering10080894

**Published:** 2023-07-28

**Authors:** Nada Rabbat, Amad Qureshi, Ko-Tsung Hsu, Zara Asif, Parag Chitnis, Seyed Abbas Shobeiri, Qi Wei

**Affiliations:** 1 Department of Bioengineering, George Mason University, Fairfax, VA 22030, USA; nmohamad@gmu.edu (N.R.); aquresh@gmu.edu (A.Q.); khsu5@gmu.edu (K.-T.H.); pchitnis@gmu.edu (P.C.); abbas.shobeiri@inova.org (S.A.S.); 2 Inova Fairfax Hospital, Fairfax, VA 22042, USA

**Keywords:** pelvic floor muscle, ultrasound imaging, deep learning, segmentation

## Abstract

Levator ani muscle (LAM) avulsion is a common complication of vaginal childbirth and is linked to several pelvic floor disorders. Diagnosing and treating these conditions require imaging of the pelvic floor and examination of the obtained images, which is a time-consuming process subjected to operator variability. In our study, we proposed using deep learning (DL) to automate the segmentation of the LAM from 3D endovaginal ultrasound images (EVUS) to improve diagnostic accuracy and efficiency. Over one thousand images extracted from the 3D EVUS data of healthy subjects and patients with pelvic floor disorders were utilized for the automated LAM segmentation. A U-Net model was implemented, with Intersection over Union (IoU) and Dice metrics being used for model performance evaluation. The model achieved a mean Dice score of 0.86, demonstrating a better performance than existing works. The mean IoU was 0.76, indicative of a high degree of overlap between the automated and manual segmentation of the LAM. Three other models including Attention UNet, FD-UNet and Dense-UNet were also applied on the same images which showed comparable results. Our study demonstrated the feasibility and accuracy of using DL segmentation with U-Net architecture to automate LAM segmentation to reduce the time and resources required for manual segmentation of 3D EVUS images. The proposed method could become an important component in AI-based diagnostic tools, particularly in low socioeconomic regions where access to healthcare resources is limited. By improving the management of pelvic floor disorders, our approach may contribute to better patient outcomes in these underserved areas.

## 1. Introduction

The levator ani muscle (LAM) is a funnel-shaped structure in the pelvic floor. It is responsible for elevating and supporting the pelvic floor, along with providing functionality during urination, defecation, and sexual function, and allows other structures to pass through it [1]. When these functions are dysregulated, women and their family units suffer physically, mentally, socially, and economically. During vaginal birth, when damage occurs to the LAM, a host of conditions classified under pelvic floor disorders (PFDs) develop. Approximately 37% of all women are affected by PFDs, with about 19% requiring surgery during their lifetime. Levator avulsion injury is a common side effect of vaginal births, which occurs in up to 35–36% of women, only after the first birth [2,3]. Some of the muscles in the pelvic floor are stretched up to three times their original length during childbirth to allow the passage of the baby, and thus, dramatic stretches can lead to levator avulsion [4]. The avulsion of the pubovisceral components of the LAM is commonly linked to pelvic organ prolapse (POP), along with other pelvic floor disorders such as urinary or fecal incontinence [5].

POP affects normal bodily function, including a downward pelvic movement that could lead to pelvic organs protruding into the vagina [3]. Although research supports that women who have given birth vaginally are more likely to suffer from more extreme prolapse, LAM avulsion can cause complications for nulliparous women as well [5]. The treatment of prolapse or avulsions usually involves complicated surgery [6]. In order to qualify for surgical treatment, an assessment of the integrity and structure of the LAM must be conducted, and operation is performed only on individuals with a smaller risk of post-surgery complications. Such an assessment is conducted using pelvic floor imaging [7].

**Figure 1:**
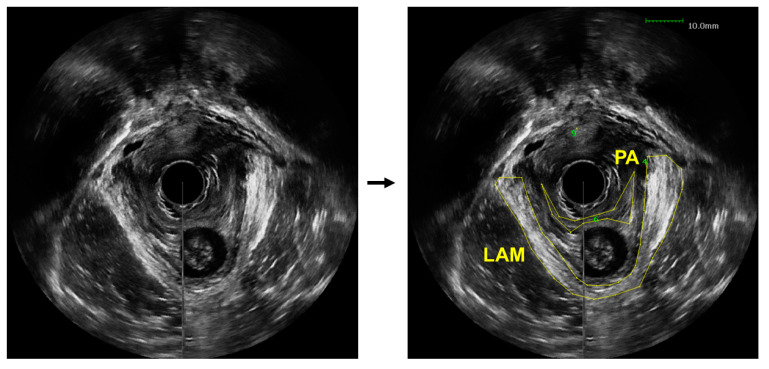
An axial view image obtained from a 3D endovaginal ultrasound image acquired from a patient with pelvic floor prolapse (**left**). Levator ani muscle (LAM) and puboanalis (PA) were traced by an urogynecologist using the BK viewer software (**right**).

The diagnosis of LAM avulsion can be performed through a medical imaging-based examination, which is subjected to challenges. While magnetic resonance imaging (MRI) provides exceptional spatial resolution and contrast, it is an expensive imaging modality that causes significant discomfort to many patients, such as claustrophobia and anxiety [7]. Ultrasound imaging is the most common diagnostic method for pelvic floor disorders, as it is still able to achieve a high contrast while maintaining a low cost and minimal discomfort for patients [8]. Then, to diagnose an avulsion, a specialized sonographer or urogynecologist would visually examine the obtained US images to identify the injury sites and assess possible LAM damage. Even with trained experts, interpretation of pelvic floor conditions from US images is challenging. Part of the reason is the limited contrast between the LAMs and the surrounding soft tissues, which makes the delineation of the LAMs challenging, especially when the LAMs are injured, as demonstrated in Figure 1. In these US images obtained from a patient with pelvic floor prolapse, the LAM and puboanalis muscle do not show obviously clear boundaries. As a result, US-based diagnosis can be subject to unavoidable operator-dependent variability. Another barrier is the significant amount of time needed to perform visual examination and comparison [9]. Instead of being able to diagnose a patient shortly after the ultrasound images are acquired, there is customarily a wait-time of a few weeks. In some cases, this prolonged diagnosis time can lead to worsening of symptoms and condition and, therefore, further discomfort for the patient in both the short and long term. To combat these clinically important issues, we propose to utilize deep learning to automatically outline the LAM from patient ultrasound data to assist clinical examination of levator avulsion and pelvic floor prolapse.

The automation of this process will help with diagnostic accuracy and consistency as well as reduce diagnostic turnover time for patients, along with providing support to underserved rural areas, where 20% of the American population resides, but only 9% of physicians serve in these areas [10]. Over 50% of rural counties do not have access to a hospital with obstetric services, indicating limited access to healthcare professionals, especially professionals with expertise in diagnosing women’s pelvic floor disorders [11]. If the process of automated LAM delineation is sufficiently accurate, it can become an important component in an AI-based diagnostic tool to be conveniently, reliably, and economically efficiently used for improved management of pelvic floor disorders in places with low resources.

There have been few studies on applying machine learning techniques to examine the LAM and POP. The most relevant work applied deep learning (DL) to perform segmentation of the LAM from 3D transperineal ultrasound images [11]. The reported segmentation result was about 0.66 in terms of Dice similarity coefficient. An automated framework to diagnose LAM avulsion from transperineal ultrasound images was developed in [7]. Convolutional neural network (CNN) models were used to predict LAM avulsion and an AUC of 0.86 was achieved based on the validation data. A novel CNN model was developed and applied to segment pelvic floor structures from magnetic resonance images (MRI). The best performance on LAM segmentation in terms of Dice similarity coefficient was reported to be 0.61. Previous work showed that deep learning is a promising technique to objectively and quantitatively examine LAM injuries and characteristics.

Inspired by these recent research works, we applied deep learning to automatically segment the LAM from 3D endovaginal ultrasound images (EVUS). Our first contribution is that this was the first time that automated LAM segmentation was performed on 3D EVUS images, which was different from previous work using transperineal US images. The second contribution is that we employed a large number of clinical EVUS images acquired from both healthy subjects and patients with pelvic floor disorders. Examination of the feasibility of using DL models on patients’ US images is important because, eventually, such models are to be used in clinics to diagnose damaged LAMs, which show significantly more complex traits than normal LAMs and are harder to segment. Three DL-based segmentation models were trained and compared to systematically examine automated EVUS segmentation, which is our third contribution. Our last contribution is that all models showed better performance than previous work.

In the following sections, we first describe the 3D EVUS data processing and then the deep learning segmentation framework. The results are then presented, followed by a discussion on what this means for future works and clinical applications. A review of the challenges encountered and directions for future improvements is also included.

## 2. Materials and Methods

### 2.1. Dataset

The ultrasound images acquired in a previously conducted work approved by the Institutional Ethics Committee (IRB) of the Inova Health System, Falls Church, VA, were analyzed retrospectively [12]. The 3D endovaginal ultrasound images were collected using a BK FlexFocus 3000 ultrasound machine, with a 16 MHz probe and a 360° rotational transducer. Unlike transperineal ultrasound (TPUS), an imaging modality in which an ultrasound probe is placed on the perineum, 3D EVUS acquires images from a probe placed in the vagina and thus provides a 360° view of the pelvic floor structures. Three-dimensional EVUS has the advantage of being noninvasive, affordable, able to provide 3D anatomical information, and fast to acquire.

**Figure 2:**
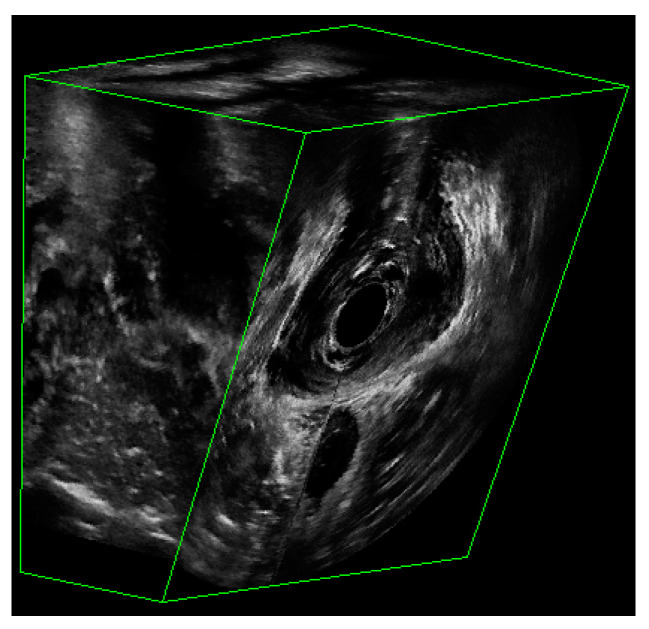
The partial view of a 3D EVUS image that shows the US signal.

The dataset consisted of 3D EVUS images from both healthy subjects as well as patients having different degrees of pelvic floor deficiency. All participants were assessed for their stages of pelvic floor support as well as their LAM deficiency and avulsion. Participants with a deficiency score less than or equal to 6 were considered to have normal LAMs, while those with a score over 6 were considered to have defective LAMs. The details about patient inclusion criteria can be found in [12]. Figure 2 shows an example of a 3D EVUS image. Each 3D EVUS image had a volume of about 94 mm by 94 mm by 64 mm. While the image dimension of each axial image was constant at 94 mm in width and height, the third dimension varied slightly from patient to patient. For the purposes of this study, the only frame of reference adopted was the axial view of the pelvic floor, in which the levator ani and other muscles were outlined by a trained technician and then reviewed. The plane containing the minimal levator hiatus (MLH) was first identified by using the volume navigation tool in the BK 3DView software specialized for the BK ultrasound machine.

**Figure 3:**
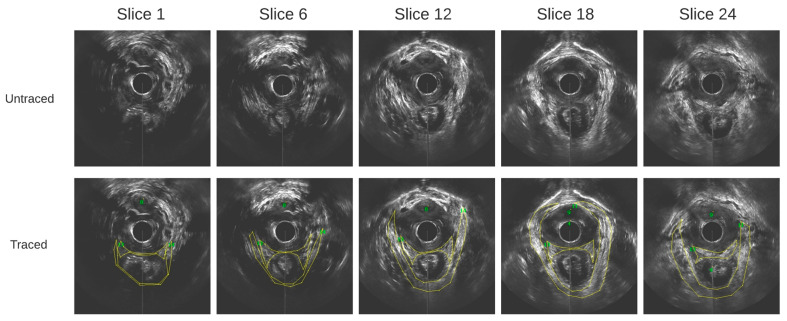
Axial ultrasound images extracted from the 3D EVUS data from one healthy participant at varying slice depths, and their corresponding traces.

Outlining was performed on contiguous axial images at 1 mm step size. The images obtained from a healthy participant at varying slice depths are shown in Figure 3. These images are all in parallel. All outlines were reviewed by a senior urogynecologist.

Both the traced and corresponding untraced images were exported for automated segmentation, resulting in a total of 1015 images in the dataset. The breakdown of the number of images in the healthy and defective LAM groups respectively is shown in Table 1.

### 2.2. Pre-Processing

**Figure 4:**
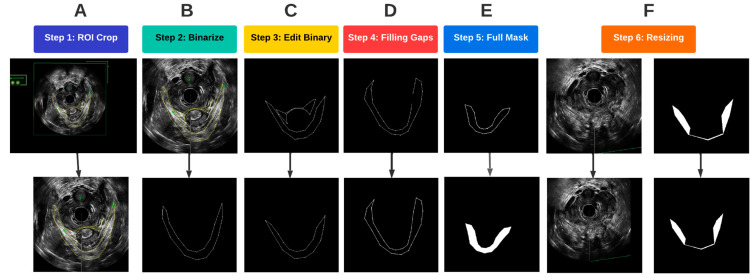
Flowchart of the image pre-processing steps.

After the traced and untraced images from the volumetric image data were obtained, several procedures were performed on the images to prepare the training data for deep learning-based segmentation. A flowchart of all the steps involved in pre-processing is depicted in Figure 4. These processes were completed in MATLAB 2022a.

A region of interest (ROI) was first determined such that it encompassed the entirety of the LAM for all traced images. To optimally prepare the images for segmentation, the probe location was used as an anchor to automatically crop and align the ROI for each of the traced and untraced images. As a result, the probe center was at the same location in each image so that the LAM location in the image was standardized (Figure 4A). Once the images were cropped and aligned, the traced images were converted to binary images, with the background being assigned to black, and any traces, which. in our case, were yellow, were assigned to the foreground in white (Figure 4B). In the original traced images, pelvic floor structures other than the LAM were also outlined for other analyses. Since the focus of this study was on segmentation of the LAM, traces of other structures, such as the puboanalis muscle, were removed (Figure 4C).

Some LAM traces had gaps along the outlined boundary, as shown in Figure 4D. Such discontinuity caused issues when defining the LAM interior regions, which required closed boundary outlines. A concave hull algorithm was applied to successfully fill in any missing pixels along the outline of the traced LAM, which provided a closed trace curve for each LAM (Figure 4D) [13]. With refined traces, image masks were constructed by assigning all pixels inside the trace as foreground, as shown in Figure 4E. The final step in pre-processing before passing the data into the deep learning models was resizing. The mask images as well as the corresponding EVUS images were padded by black pixels at the right and bottom edges to create images with a size of 512 × 512, which is an image size compatible with the deep learning segmentation procedure (Figure 4F).

### 2.3. Training and Testing Data Preparation

The axial images were separately into a training set to train the DL models and another test set to assess model performance. Since defective LAMs have different anatomical characteristics from normal LAMs, we needed to make sure the training and test sets contain good representations of each category. The images of normal LAMs were randomly split into a training set, containing 85% of the images, while the remaining 15% became the test set. The same procedure was performed for the images of defective LAMs. The two training sets were then combined to train the DL models, while the combined test set was used for evaluation. Such a randomization and splitting procedure ensured that both the training set and the test set contained the same proportion of normal cases and defective cases to avoid possible bias due to the difference of the two types. Table 2 summarizes the number of images in the training and test sets.

### 2.4. DL Model Configuration

Muscle segmentation is a common task performed in a plethora of previous works, providing ample data on the benefits of each approach. In most cases that involve segmentation, the boundaries of structures are fairly clear, as is the case for left ventricle, liver, and bony orbit segmentations [14,15,16]. Usually, these segmentations rely on MRI or computed tomography imaging but undergo similar methods for segmentation using ultrasound images. Clear boundaries certainly are more advantageous because they provide more image features for automated segmentation methods to recognize. In the case of pelvic floor muscle segmentation using ultrasound images, the task is much harder. The LAM and surrounding soft tissues share similar image intensity, which results in blurry LAM boundaries.

There are mainly two divisions of studies conducted to automatically segment pelvic floor anatomical structures. Some studies dealt with the segmentation of a more generalized pelvic floor area, and others segmented specific pelvic floor structures. For the former, the levator hiatus is usually the segmentation target, whose boundaries are far clearer than the LAM. Bonmati et al. implemented a convolutional neural network (CNN) using a novel nonlinear self-normalizing activation function and obtained a levator hiatus segmentation Dice similarity coefficient of 0.9 from 91 oblique axial ultrasound images [9]. Another study implemented a U-net architecture with dense connections to concatenate feature maps from coarse to fine layers [17]. The image dataset was accompanied by a binary mask of the levator hiatus, which was constructed by three different physicians, and averaged to form one ground truth mask. A level-set method was also employed in post-processing, which improved the outline of the levator hiatus.

In the case of the second division of studies, the segmentation tasks were performed using MRI or transperineal ultrasound images and different automatic segmentation models were implemented. One project utilized a CNN with multi-resolution feature pyramid (MRFP) layers to segment the uterus, rectum, bladder, and LAM using MRI images [18]. Each MRFP module was made up of four dilated convolution layers and one average pooling layer. To deal with segmentation failures, another post-processing step was added, a level set, which was represented by a partial differential equation. This involved the initial computation of the minimum 3D boundary of the organ extracted by the CNN and cropping, with the level set then applied to the cropped image. The results were only used, instead of the initial segmentation, if the Dice similarity coefficient was improved. However, the training time was around 50 h per network, which is too slow for clinical applications. Another study implemented active appearance models to segment the puborectalis muscle using 3D transperineal ultrasound images [19]. Lastly, a novel recurrent 2D U-Net with some convolution layers replaced by conventional long short-term memory cells, which were set to remember certain features of the data and “forget” others, was developed and applied to perform LAM segmentation using transperineal US images [20]. This model has the advantage of preserving GPU memory, which is a major constraint when segmenting 3D ultrasound data.

The metric results of the previous work in the literature indicated that CNN is a good candidate to segment the LAM using MRI or transperineal ultrasound images. Although our 3D EVUS images are different from the MRI or transperineal US images used in previous works, our segmentation goal is the same. A U-Net architecture and two of its variations were adapted for our study. U-Net is a convolutional neural network with an altered architecture [21]. One U-Net model used in our study replaces the up-sampling layer in a CNN with a pooling one. The second model used in our study is the attention U-Net model, which implements attention gates to the encoding path to focus on the target features of the images [22]. Models trained with attention mechanisms are capable of implicitly learning to disregard irrelevant regions in an input image, while simultaneously emphasizing salient features that are relevant to a specific task. The third model is the FD-UNet, which uses dense connectivity to contract and expand the paths of the network [23]. By connecting every neuron in one layer to every neuron in the previous layers, dense connectivity enables the network to capture complex, non-linear relationships between the input and output data. Dense connectivity can also lead to more accurate predictions and better generalization of the network to new data when properly regularized, thus essentially enhancing information flow. The last model implemented is the Dense-UNet, which connects the convolution block outputs to the inputs of all subsequent blocks. This allows feature reuse and alleviates the vanishing gradient problem [24]. Although the FD-UNet uses dense layers based on the Dense-UNet, we included the latter one in our study to maintain a fair comparison with the other methods.

TensorFlow was used to implement the four models. The models were tested using a Lambda Vector workstation with an Intel Core i9-10980XE 3.00 GHz GPU and NVIDIA RTX A5000 GPU. Each model was trained over 50 epochs, with a batch size of 16. The trained models can be downloaded from our GitHub repository (https://github.com/AAQ2017/LAMSegment, accessed 13 April 2023). Table 3 shows the DL model architecture parameters, including the number of epochs, activation function, loss function, and optimizer.

### 2.5. Evaluation Metrics of the Methods

To assess the segmentation effectiveness of the DL models, the commonly used Jaccard Mean Intersection-Over-Union (IoU) and the Mean Dice Similarity Coefficient (F1) were used to measure the pixel-wise overlap between the predicted mask and the ground truth. These scores typically range from 0 to 1, with a score of 0 indicating there is no overlap between the predicted LAM mask and the ground truth mask, whereas a score of 1 indicates that the true mask is perfectly predicted. Equations (1) and (2) describe the formulae for estimating the IoU and Dice Similarity Coefficient:

(1) $$\mathrm{IoU}=\frac{TP}{TP+FP+FN}$$

(2) $$\mathrm{Dice}=\frac{2\times TP}{\left(TP+FP\right)+(TP+FN)}$$

where true positive (TP) represents the number of pixels that are accurately predicted while simultaneously being present in the ground truth mask; false positive (FP) refers to the predicted pixels that are not in the ground truth mask; and false negative (FNs) represents the incorrectly classified pixels compared to the ground truth.

## 3. Results

We first examined the performance of the four models using 1015 EVUS images containing both healthy and defective LAMs. Each model was trained on 862 images with the outlined masks. They were then tested on a separate set of 153 US images. It took about 15 min to train the U-Net model. The attention U-Net, FD-UNet, and Dense-UNet models had a training time of 23 min, 37 min, and 102 min, respectively.

The metric results are summarized in Table 4. All models were able to achieve an IoU of 0.74 or higher, and a Dice score of 0.84 or higher, on the test data, demonstrating the efficacy of the method in automatically outlining the LAM on the EVUS images. The training accuracies showed an IoU score of at least 0.84 and a Dice score of at least 0.91, which were higher than the testing accuracies, as expected. One-way ANOVA tests were performed using the IoU and Dice scores of the three models. No statistically significant difference was found among the results of the models (*p*-value = 0.15 for IoU and *p*-value = 0.10 for Dice).

In addition to the IoU and Dice scores, sensitivity, specificity, false positive (FP) rate, and false negative (FN) rate were also computed to further compare the models, and the results are presented in Table 5. The FD-UNet model had the highest sensitivity while the Dense-UNet model had the lowest. All models showed reasonably good sensitivity above 0.82, demonstrating their efficacy in segmentation.

**Figure 5:**
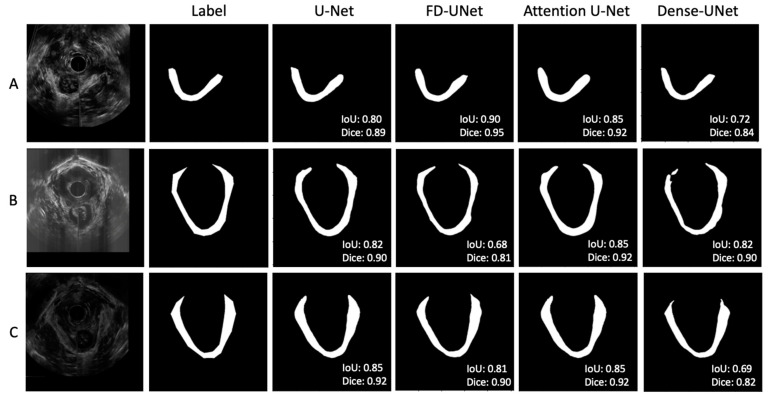
(**A**–**C**) showed three sets of example results from different test images. In general, agreement between the manual label and the predicted outputs was observed. Through visual inspection, the models seem to perform similarly, except for the corners of the LAM.

Figure 5 shows three sets of examples based on the outputs from the DL models. The DL-generated LAM boundary seems to be smoother than that of manual tracing, which is anatomically more accurate. The FD-UNet model was able to locate most of the LAM but lacked precision at the top right area, as shown in Figure 5A. The Dense-UNet model did a less satisfying job in delineating the LAM. Through a visual examination of all testing image results, in most cases, all four models were able to segment the LAM reasonably well, with similar results, as shown in Figure 5. Figure 5B demonstrates a less optimal result, which shows some disparity between the predicted outcome and the traced region, and this result is explained in the discussion section. These less successful cases appear in only a small number of testing images.

For clinicians who use EVUS images for diagnosis via visual examination, images containing defective LAMs are harder to interpret because of the variable muscle anatomy due to deficits. We examined whether it was also the case for the DL models, in which automated segmentation of healthy LAMs is easier and thus more accurate. Another U-Net model was trained on 434 EVUS images containing healthy LAMs and then tested on 77 test images of healthy LAMs. Surprisingly, we did not see an improved performance when only healthy LAM images were included (Table 6). The results are encouraging because it shows that the DL models can segment defective LAMs as accurately as healthy LAMs. If the automated segmentation framework is applied clinically, many of the examined LAMs would be subject to some deficits and variability, rather than being completely normal. It is important to develop and use DL models that are robust to handle LAM abnormality.

Data augmentation is commonly used in sematic image segmentation to increase the number of training data as well as to present input variety to the classifier, aiming to achieved improved segmentation results [24]. We examined whether data augmentation would result in more accurate LAM segmentation. Spatial transformation-based augmentation is not applicable to our data because these axial images have already been translated and aligned to have the same probe location and, thus, similar LAM location. Instead, we performed intensity-based augmentation. Contrast adjustment at 0.8, 0.9, 1.1, and 1.2 as well as brightness adjustment at −0.1 and 0.1 were utilized to simulate image variability. Images containing healthy LAMs were used in training and testing. The results are summarized in Table 7. Comparing the IoU and Dice scores in Table 7 to those in Table 6, we found that data augmentation significantly improved the training accuracy. However, it did not affect the testing accuracy.

**Figure 6:**
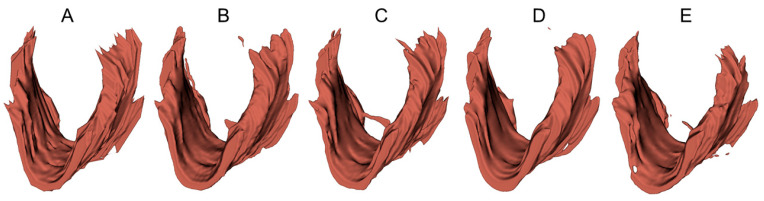
Three-dimensional surfaces of the LAM reconstructed from (**A**) manual segmented traces and (**B**–**E**) LAM boundaries segmented by U-Net, FD-UNet, attention U-Net, and Dense-UNet respectively.

To provide a visual demonstration of the segmentation results in three dimensions, 3D surfaces were reconstructed in 3D Slicer (http://www.slicer.org, accessed on 12 December 2022) using the LAM delineations obtained from an example 3D EVUS image in the test set. A smooth factor of 0.5 was applied. As shown in Figure 6, all models were able to locate the LAM in the volumetric US image and depict the shape of the LAM. However, obvious discrepancies near the upper attachments exist, consistent with the 2D segmentation accuracy assessment reported in Table 4 and Table 5.

## 4. Discussion

Our results demonstrate the feasibility of applying a deep learning method to perform automated LAM segmentation. This is the first time that such an analysis was performed on a large number of images extracted from 3D EVUS data. Through a visual inspection of the predicted LAM masks in both the training and testing sets, the DL models seems to have performed an adequate job segmenting the LAM.

As previously mentioned, the main dataset consists of both normal and defective LAMs traced by a trained technician. The models were able to achieve a reasonably high mean IoU and Dice scores with this dataset, as shown in Table 3. An alternate dataset, which consists of the same traced data used in this study, along with an additional set of images traced by novice technicians, was also experimented to train and test a U-Net model. The results for this enlarged set of data were far worse, with the IoU and Dice scores slightly above 0.5. After examining the traces performed by the novice technicians, we found significant errors made by the novice technicians, which explained the errors made by the DL models. This stresses the importance of using high-quality labeled data for training DL models; otherwise, they would be taught in the wrong way. We also noticed that there were a small number of cases that the DL models generated segmentation containing separate regions, as seen in Figure 5B. This happened either near the attachments of LAM or on the images of defective LAMs, which are much thinner and have poorer contrast than normal LAMs and, thus, posing challenges to producing a continuous LAM region. Considering the inherent challenge of segmenting the LAM using US images, such variable results are expected.

When compared to other published studies, specifically those related to LAM segmentation, U-Net and its variations show more promising results. The proposed models were able to achieve a mean Dice score of at least 0.84 and a mean IoU of at least 0.69, while other methods used for segmenting the LAM achieved a mean Dice score around 0.60–0.65, as shown in Table 8. These comparative results are important, as our automated segmentation procedure is one of the few dealing with LAM segmentation, and, to our knowledge, the only one performing segmentation using endovaginal ultrasound images. These results indicate that the proposed DL-based segmentation method is a viable option for clinical diagnosis. It is also important to note that the number of images in our dataset is large, showing the generalizability of the method in examining different pelvic floor conditions. The only other work with more images in the training set was tasked with segmenting the levator hiatus [17], a much larger area with more prominent boundaries.

Our proposed models also performed slightly better when both normal and defective LAM ultrasound images traced by a trained technician were used, instead of including only the normal subset of data. We experimented with commonly used image augmentation techniques, including contrast and brightness adjustment, but did not observe an improved model performance. The ineffectiveness of data augmentation indicates the inherent challenges of segmenting the LAM using EVUS images as well as the adequate variation in the training data.

Results of comparable accuracy are expected when the trained models are applied to the US images acquired from other EVUS machines. The reason is that our training images already contain US images of varying contrast and brightness, which were adjusted by a sonographer for optimal visualization of the anatomical structure of interest in each EVUS volume. Therefore, the variability associated with the training data shows the generalizability of the trained model in terms of its application to future US image data.

While this segmentation task was straightforward, there were many hurdles to overcome. Since the data was not perfectly curated to segment the LAM, during the binarization of the masks, many images containing secondary structures, as previously mentioned, had to be manually excluded. Unfortunately, for a dataset as large as the one acquired, the manual editing of each image proved very time consuming. Any additional data to be added to train the model would most likely have to undergo the same tedious manual editing unless a more specific manual tracing protocol is to be executed.

There are steps that can be taken to possibly improve segmentation results in the future. To start, adding more training images would be helpful in improving evaluation metrics. Averaging the traces performed by multiple technicians could also help improve the accuracy of the training mask and, therefore, improve IoU and Dice. This would also reduce variability in the test results based on specific technician styles, as the model used should be trained according to traces made by multiple professionals. In the future, an additional step in the model to measure displacement, size, thickness, and other muscle metrics might help draw conclusions on the nature of the muscle of interest (damaged vs. normal), and extend the application of the model as a classification tool able to diagnose a LAM avulsion. This can be achieved by expanding on previous works performing such tasks. One such study collected data that consisted of both patients with and without a levator ani muscle avulsion diagnosis using transperineal ultrasound [7]. The images were collected at rest, during pelvic floor muscle contraction, and on Valsalva. This model can be used to expand our own and make the diagnostic processes even simpler.

## 5. Conclusions

This study aimed to address the gap in existing literature with regard to segmentation tasks pertaining to 3D endovaginal ultrasound images obtained from patients with LAM avulsion and other deficits. Previous studies performed segmentation procedures using transperineal ultrasound (TPUS) and neglected the potential of EVUS, which is a comparable modality to MRI. We conducted a study to test four CNN segmentation models, based on the U-Net architecture, on clinically acquired EVUS images to determine the feasibility of extending automated segmentation to AI-assisted modality for image-based diagnosis.

Our findings demonstrate that the U-Net segmentation model employed in this study outperformed the existing approaches reported in the literature in terms of average IoU (0.76) and Dice (0.86) metrics. This suggests that U-Net models have the potential to outline the LAM, thereby improving the diagnostic capabilities of DL-based methods for pelvic floor disorders.

By showing the efficacy of the DL-based segmentation methods using EVUS images for LAM segmentation, our study enables researchers to expand the scope of automated imaging analysis methods for the diagnosis of pelvic floor disorders. These results also provide a basis for further research and development of AI-assisted diagnostic frameworks to enable efficient and accurate management of pelvic floor disorders.

## Figures and Tables

**Table 1:** Summary of number of participants and axial images included in the analysis.

	Number of Participants	Number of Images	Percentage
Healthy	14	504	50.3%
Defective	13	511	49.7%
**Total**	27	1015	100%

**Table 2:** Number of images allocated for training and testing the DL models.

	Healthy LAM	Defective LAM	Total	Percentage
Number of Images in Training Set	434	428	862	85%
Number of Images in Test Set	77	76	153	15%
**Total Number of Images**	511	504	1015	100%

**Table 3:** Architecture parameters of the four DL models tested.

	U-Net	Attention U-Net	FD-UNet	Dense-UNet
Epochs	50	50	50	50
Activation Function	ReLU and Softmax	ReLU, Softmax, and Sigmoid	ReLU and Softmax	ReLU and Softmax
Loss Function	Categorical Loss Entropy	Categorical Loss Entropy	Categorical Loss Entropy	Categorical Loss Entropy
Optimizer	Adam	Adam	Adam	Adam

**Table 4:** Segmentation performance of U-Net, attention U-Net, FD-UNet, and Dense-UNet based on EVUS images containing healthy and defective cases. The IoU and Dice scores were computed for the LAM. No augmentation was performed. SD: standard deviation.

		Training Accuracy (N = 862)	Testing Accuracy (N = 153)
**U-Net**	**IoU (mean** **± SD)**	0.86 ± 0.06	0.76 ± 0.11
	**Dice (mean** **± SD)**	0.92 ± 0.04	0.86 ± 0.08
**Attention U-Net**	**IoU (mean** **± SD)**	0.84 ± 0.07	0.75 ± 0.11
	**Dice (mean** **± SD)**	0.91 ± 0.05	0.85 ± 0.08
**FD-UNet**	**IoU (mean** **± SD)**	0.84 ± 0.09	0.74 ± 0.12
	**Dice (mean** **± SD)**	0.91 ± 0.06	0.84 ± 0.10
**Dense-UNet**	**IoU (mean** **± SD)**	0.78 ± 0.11	0.69 ± 0.13
	**Dice (mean** **± SD)**	0.87 ± 0.08	0.81 ± 0.10

**Table 5:** Segmentation performance of U-Net, attention U-Net, FD-UNet, and Dense-UNet on the testing images (N = 153). Sensitivity, specificity, false positive (FP) rate, and false negative (FN) rate were computed for the LAM. No augmentation was performed. SD: standard deviation.

	Sensitivity (Mean ± SD)	Specificity (Mean ± SD)	FP Rate (Mean ± SD)	FN Rate (Mean ± SD)
**U-Net**	0.84 ± 0.10	0.99 ± 0.00	0.01 ± 0.00	0.16 ± 0.10
**Attention U-Net**	0.84 ± 0.11	0.99 ± 0.01	0.01 ± 0.01	0.16 ± 0.11
**FD-UNet**	0.89 ± 0.09	0.99 ± 0.11	0.01 ± 0.01	0.11 ± 0.09
**Dense-UNet**	0.82 ± 0.13	0.99 ± 0.01	0.01 ± 0.01	0.18 ± 0.13

**Table 6:** Segmentation performance of U-Net using EVUS images containing only healthy cases. The IoU and Dice scores were computed for the LAM. No augmentation was performed. SD: standard deviation.

	Training Accuracy (N = 434)	Testing Accuracy (N = 77)
**IoU (mean** **± SD)**	0.85 ± 0.07	0.73 ± 0.13
**Dice (mean** **± SD)**	0.91 ± 0.04	0.83 ± 0.10

**Table 7:** Segmentation performance of U-Net using EVUS images containing only healthy cases. The IoU and Dice scores were computed for the LAM. Image augmentation was performed. SD: standard deviation.

	Training Accuracy (N = 434)	Testing Accuracy (N = 77)
**IoU (mean ± SD)**	0.96 ± 0.02	0.73 ± 0.13
**Dice (mean ± SD)**	0.98 ± 0.01	0.84 ± 0.10

**Table 8:** Metric results of ours and other similar models in the literature.

Study	Imaging Modality	Segmentation Region	Number of Images	Segmentation Method	Mean Dice	Mean IoU
Ours	EVUS	LAM	1015	U-Net, Attention U-Net, FD-Unet, and Dense-UNet	>0.84	>0.69
Noort et al. [20]	TPUS	LAM	100	Recurrent U-Net	0.65	-
Feng et al. [18]	MRI	LAM	528	CNN + MRFP	~0.61	-
Noort et al. [19]	TPUS	Puborectalis	50	Active appearance model	~0.6	-
Bonmati et al. [9]	TPUS	Levator Hiatus	91	CNN	~0.90	~0.82
Li et al. [17]	2D US	Levator Hiatus	1130	Dense U-Net	~0.96	~0.95
Noort et al. [25]	TPUS	Urogenital Hiatus	373	CNN	0.94	-

## Data Availability

A GitHub repository that contains the trained DL models has been created and can be assessed using the following link: https://github.com/AAQ2017/LAMSegment, accessed 13 April 2023.

## References

[1] Gowda S.N., Bordoni B. (2023). Anatomy, Abdomen and Pelvis: Levator Ani Muscle. StatPearls.

[2] Dietz H.P., Moegni F., Shek K.L. (2012). Diagnosis of levator avulsion injury: A comparison of three methods: Diagnosis of levator avulsion. Ultrasound Obstet. Gynecol..

[3] Lammers K., Prokop M., Vierhout M.E., Kluivers K.B., Fütterer J.J. (2013). A pictorial overview of pubovisceral muscle avulsions on pelvic floor magnetic resonance imaging. Insights Imaging.

[4] Das S., Hansen H.H., Hendriks G.A., Noort F.V.D., Manzini C., van der Vaart C.H., de Korte C.L. (2021). 3D Ultrasound Strain Imaging of Puborectalis Muscle. Ultrasound Med. Biol..

[5] Nygaard I., Barber M.D., Burgio K.L., Kenton K., Meikle S., Schaffer J., Spino C., Whitehead W.E., Wu J., Brody D.J. (2008). Prevalence of Symptomatic Pelvic Floor Disorders in US Women. JAMA.

[6] Wong N.K.L., Cheung R.Y.K., Lee L.L., Wan O.Y.K., Choy K.W., Chan S.S.C. (2021). Women with advanced pelvic organ prolapse and levator ani muscle avulsion would significantly benefit from mesh repair surgery. Ultrasound Obstet. Gynecol..

[7] Wu S., Ren Y., Lin X., Huang Z., Zheng Z., Zhang X. (2022). Development and validation of a composite AI model for the diagnosis of levator ani muscle avulsion. Eur. Radiol..

[8] Baușic A., Coroleucă C., Coroleucă C., Comandașu D., Matasariu R., Manu A., Frîncu F., Mehedințu C., Brătilă1 E. (2022). Transvaginal Ultrasound vs. Magnetic Resonance Imaging (MRI) Value in Endometriosis Diagnosis. Diagnostics.

[9] Bonmati E., Hu Y., Sindhwani N., Dietz H.P., D’Hooge J., Barratt D., Deprest J., Vercauteren T. (2018). Automatic segmentation method of pelvic floor levator hiatus in ultrasound using a self-normalizing neural network. J. Med. Imaging.

[10] Rosenblatt R.A. (2000). Physicians and rural America. West. J. Med..

[11] Warshaw R. Health Disparities Affect Millions in Rural U.S. Communities. *Association of American Medical Colleges*; 31 October 2017. https://www.aamc.org/news-insights/health-disparities-affect-millions-rural-us-communities.

[12] Asif Z., Tomashev R., Peterkin V., Wei Q., Alshiek J., Yael B., Shobeiri S.A. (2022). Levator ani muscle volume and architecture in normal vs. muscle damage patients using 3D endovaginal ultrasound: A pilot study. Int. Urogynecol. J..

[13] Bodhisattwa Chakraborty. Concave_Hull_Generation. 27 June 2017. https://github.com/bodhisattwa-chakraborty/Concave_Hull_Generation.

[14] Avendi M., Kheradvar A., Jafarkhani H. (2016). A combined deep-learning and deformable-model approach to fully automatic segmentation of the left ventricle in cardiac MRI. Med. Image Anal..

[15] Li W., Jia F., Hu Q. (2015). Automatic Segmentation of Liver Tumor in CT Images with Deep Convolutional Neural Networks. J. Comput. Commun..

[16] Hamwood J., Schmutz B., Collins M.J., Allenby M.C., Alonso-Caneiro D. (2021). A deep learning method for automatic segmentation of the bony orbit in MRI and CT images. Sci. Rep..

[17] Li X., Hong Y., Kong D., Zhang X. (2019). Automatic segmentation of levator hiatus from ultrasound images using U-net with dense connections. Phys. Med. Biol..

[18] Feng F., Ashton-Miller J.A., DeLancey J.O.L., Luo J. (2020). Convolutional neural network-based pelvic floor structure segmentation using magnetic resonance imaging in pelvic organ prolapse. Med. Phys..

[19] Noort F.V.D., Grob A.T.M., Slump C.H., Van Der Vaart C.H., Van Stralen M. (2018). Automatic segmentation of puborectalis muscle on three-dimensional transperineal ultrasound. Ultrasound Obstet. Gynecol..

[20] van den Noort F., Sirmacek B., Slump C.H. Recurrent U-Net for Automatic Pelvic Floor Muscle Segmentation on 3D Ultrasound. *arXiv* 29 July 2021. http://arxiv.org/abs/2107.13833.

[21] Ronneberger O., Fischer P., Brox T. U-Net: Convolutional Networks for Biomedical Image Segmentation. *arXiv* 18 May 2015. http://arxiv.org/abs/1505.04597.

[22] Oktay O., Schlemper J., Folgoc L.L., Lee M., Heinrich M., Misawa K., Mori K., McDonagh S., Hammerla N.Y., Kainz B. (2018). Attention U-Net: Learning Where to Look for the Pancreas. arXiv.

[23] Guan S., Khan A.A., Sikdar S., Chitnis P.V. (2020). Fully Dense UNet for 2D Sparse Photoacoustic Tomography Artifact Removal. IEEE J. Biomed. Health Inform..

[24] Chaitanya K., Karani N., Baumgartner C.F., Erdil E., Becker A., Donati O., Konukoglu E. (2021). Semi-supervised task-driven data augmentation for medical image segmentation. Med. Image Anal..

[25] Noort F.v.D., Manzini C., van der Vaart C.H., van Limbeek M.A.J., Slump C.H., Grob A.T.M. (2022). Automatic identification and segmentation of slice of minimal hiatal dimensions in transperineal ultrasound volumes. Ultrasound Obstet. Gynecol..

